# Prenatal testosterone triggers long-term behavioral changes in male zebra finches: unravelling the neurogenomic mechanisms

**DOI:** 10.1186/s12864-021-07466-9

**Published:** 2021-03-06

**Authors:** Alexandra B. Bentz, Chad E. Niederhuth, Laura L. Carruth, Kristen J. Navara

**Affiliations:** 1 Department of Biology, Indiana University, Bloomington, IN 47405 USA; 2 Center for the Integrative Study of Animal Behavior, Indiana University, Bloomington, IN 47405 USA; 3 Poultry Science Department, University of Georgia, Athens, GA 30602 USA; 4 Department of Plant Biology, Michigan State University, East Lansing, MI 48823 USA; 5 Neuroscience Institute, Georgia State University, Atlanta, GA 30303 USA

**Keywords:** Yolk testosterone, Maternal effect, DNA methylation, Aggression, Hypothalamus, Somatostatin, Glutamate, ADCY2, PCSK2

## Abstract

**Background:**

Maternal hormones, like testosterone, can strongly influence developing offspring, even generating long-term organizational effects on adult behavior; yet, the mechanisms facilitating these effects are still unclear. Here, we experimentally elevated prenatal testosterone in the eggs of zebra finches (*Taeniopygia guttata*) and measured male aggression in adulthood along with patterns of neural gene expression (RNA-seq) and DNA methylation (MethylC-Seq) in two socially relevant brain regions (hypothalamus and nucleus taenia of the amygdala). We used enrichment analyses and protein-protein interaction networks to find candidate processes and hub genes potentially affected by the treatment. We additionally identified differentially expressed genes that contained differentially methylated regions.

**Results:**

We found that males from testosterone-injected eggs displayed more aggressive behaviors compared to males from control eggs. Hundreds of genes were differentially expressed, particularly in the hypothalamus, including potential aggression-related hub genes (e.g., brain derived neurotrophic factor). There were also enriched processes with well-established links to aggressive phenotypes (e.g., somatostatin and glutamate signaling). Furthermore, several highly connected genes identified in protein-protein interaction networks also showed differential methylation, including adenylate cyclase 2 and proprotein convertase 2.

**Conclusions:**

These results highlight genes and processes that may play an important role in mediating the effects of prenatal testosterone on long-term phenotypic outcomes, thereby providing insights into the molecular mechanisms that facilitate hormone-mediated maternal effects.

**Supplementary Information:**

The online version contains supplementary material available at 10.1186/s12864-021-07466-9.

## Background

An individual’s phenotype can be strongly impacted by the phenotype or environment of its mother [1]. Specifically, hormone-mediated maternal effects, in which maternally derived hormones influence offspring phenotype, have been extensively studied in avian species and a primary focus of this work has been on maternal androgens. Females experiencing more social competition tend to allocate more testosterone (T) to developing offspring through egg yolks [2–8] and these offspring display increased aggression well into adulthood [8–11]. Despite the potentially adaptive benefits of generating more aggressive offspring in more competitive environments, the underlying neural mechanisms by which prenatal hormones generate lasting behavioral change is still unclear [12]. Past work has examined the role the androgen receptor plays in mediating maternal effects [13]; however, aggression is regulated by a variety of neural genes expressed across numerous pathways [14]. Genome-wide approaches would therefore help to clarify the potentially diverse mechanisms underlying the pleiotropic effects of prenatal T on complex behavioral traits.

The enduring effects of perinatal experiences on gene expression can often be linked to epigenetic gene regulation [15]. DNA methylation, for example, acts by adding methyl groups to cytosines at CpG dinucleotides, which can suppress gene expression [16]. Methylation patterns are established in early development and may be one way for hormone-induced changes to last into adulthood*.* Examples of this are evident in the methylation patterns produced by steroid-mediated sex differentiation in mammals [17] and fetal exposure to endocrine disrupters [18]. Thus far, candidate-gene analyses suggest that variation in the maternal environment can result in altered DNA methylation patterns in juvenile birds [19, 20]. However, the proposed mechanism, starting with prenatal T and leading to long-term phenotypic changes in adults, along with the potential intervening transcriptomic/epigenomic steps, has yet to be tested.

Here, we explore the lasting effects of prenatal T on adult behavioral plasticity and genome-wide patterns of gene expression and methylation in male zebra finches (*Taeniopygia guttata*). Zebra finches are a good study system because they have numerous genes that are potential targets of epigenetic regulation [21]. Zebra finch embryos also express steroid receptors during early development (prior to endogenous T production) [22]. We injected zebra finch eggs with T or a vehicle control the morning eggs were laid. Embryos are in the earliest stages of development at this point [23, 24] and, based on work in other altricial songbirds [25] and more well-known mammalian models [26], injections should coincide with a time just prior to when de novo methylation patterns are starting to become established. We measured aggression in sexually mature adult males. Then, using transcriptome profiling (RNA-Seq) and whole-genome bisulfite sequencing (MethylC-Seq), we examined patterns of gene expression and DNA methylation in steroid-sensitive brain regions containing the vertebrate social behavior network, the hypothalamus (HYPO) and nucleus taenia of the amygdala (TnA) [27, 28]. The TnA is involved in social arousal and responses to same-sex conspecifics in songbirds [29], while the HYPO plays a central role in neuroendocrine function, regulating many of the neurotransmitters and hormones associated with aggression [14, 30]. Overall, this study provides novel insight into whether prenatal T can generate lasting changes in behavior alongside altered gene expression and methylation.

## Results

### Effect of prenatal testosterone on adult aggression and plasma testosterone

Eggs from 20 breeding pairs (*n* = 109 eggs) were injected with T or a vehicle control. Overall, 54 eggs hatched (24 were males; *n* = 8 control and 16 T), yielding a hatching success of 49.5%, similar to non-manipulated eggs in captive-breeding colonies (48%) [31]. There was no significant difference in hatching success by treatment (β = − 0.48, − 1.26-0.29 95% CI; *F*_1,106_ = 1.54, *p* = 0.221) or laying order (β = 0.03, − 0.30-0.37 95% CI; *F*_1,106_ = 0.04, *p* = 0.842). There was also no significant difference in mass at hatching by treatment (β = 0.08, − 0.11-0.26 95% CI; *F*_1,18.5_ = 0.67, *p* = 0.423) or laying order (β = − 0.002, − 0.08-0.08 95% CI; *F*_1,15.3_ < 0.01, *p* = 0.957).

**Fig. 1:**
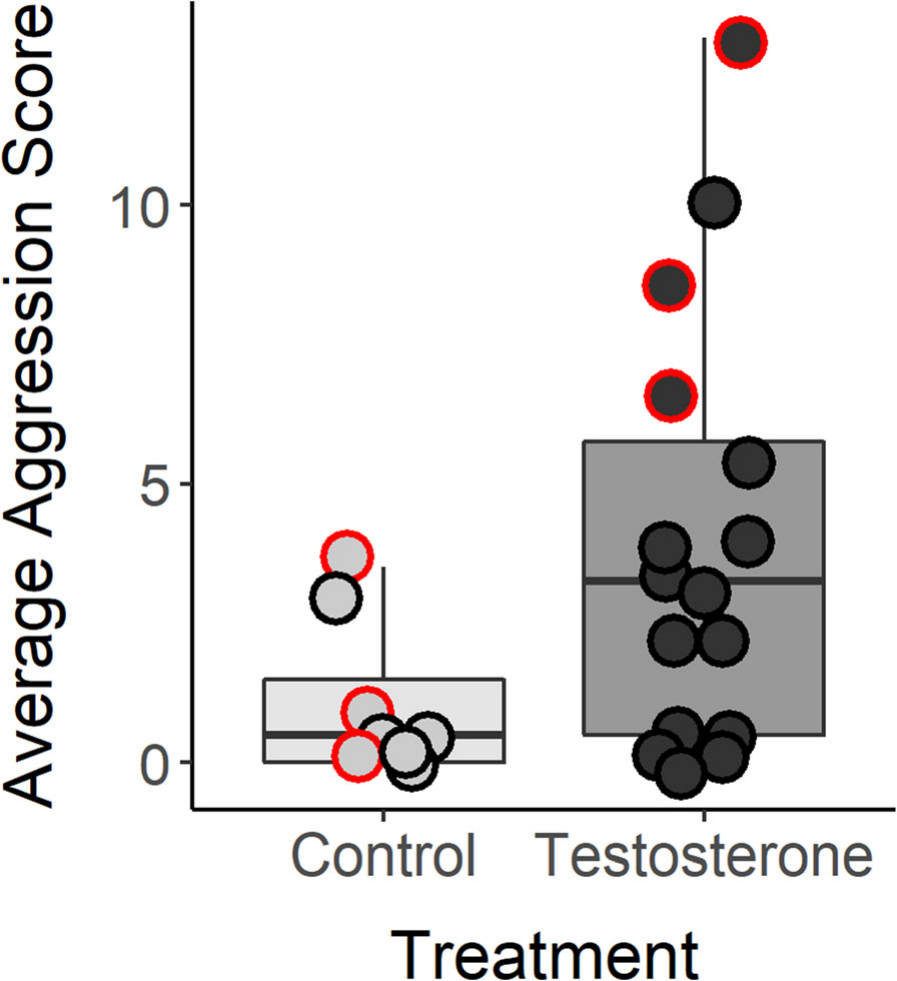
Average aggression scores by prenatal treatment. Boxplots depict the median count (horizontal line) bounded by the upper and lower quartile of aggression scores for each treatment, and whiskers represent 1.5 inter-quartile ranges. A red border identifies the three individuals per treatment that were collected for gene expression and methylation analyses

Aggression was assayed in 15 min same-sex conspecific intrusion trials in adult males (mean age = 138 days post-hatch ±14 SE), during which the number of aggressive actions performed by the subject toward the intruder were recorded. Two assays were performed over separate days (mean = 48 days apart ±5 SE) and aggression scores were averaged. Repeatability of aggression was significant across the two behavioral trials (*R* = 0.64 ± 0.18 SE; *p* = 0.005), suggesting individuals were moderately consistent in the way they responded to conspecific intruders. Males from T-injected eggs displayed more aggressive behaviors than controls (β = 0.76, 0.08–1.44 95% CI; *F*_1,21_ = 4.53, *p* = 0.045; Fig. 1); laying order was not significantly related to aggression (β = 0.12, − 0.18-0.43 95% CI; *F*_1,21_ = 0.59, *p* = 0.450). We collected trunk blood and brain tissue from unrelated males from each treatment group (*n* = 3/treatment) at the completion of the behavioral assays. Plasma T measured in trunk blood did not significantly differ by prenatal treatment (β = 0.07, − 2.59-2.45 95% CI; *t*_2.4_ = − 0.10, *p* = 0.926; T treated: 2.12 ng/mL ± 0.19 SE; control: 2.05 ± 0.65 SE), although this finding should be interpreted cautiously as the power to detect statistical significance was low.

### Identification of differentially expressed genes

**Fig. 2:**
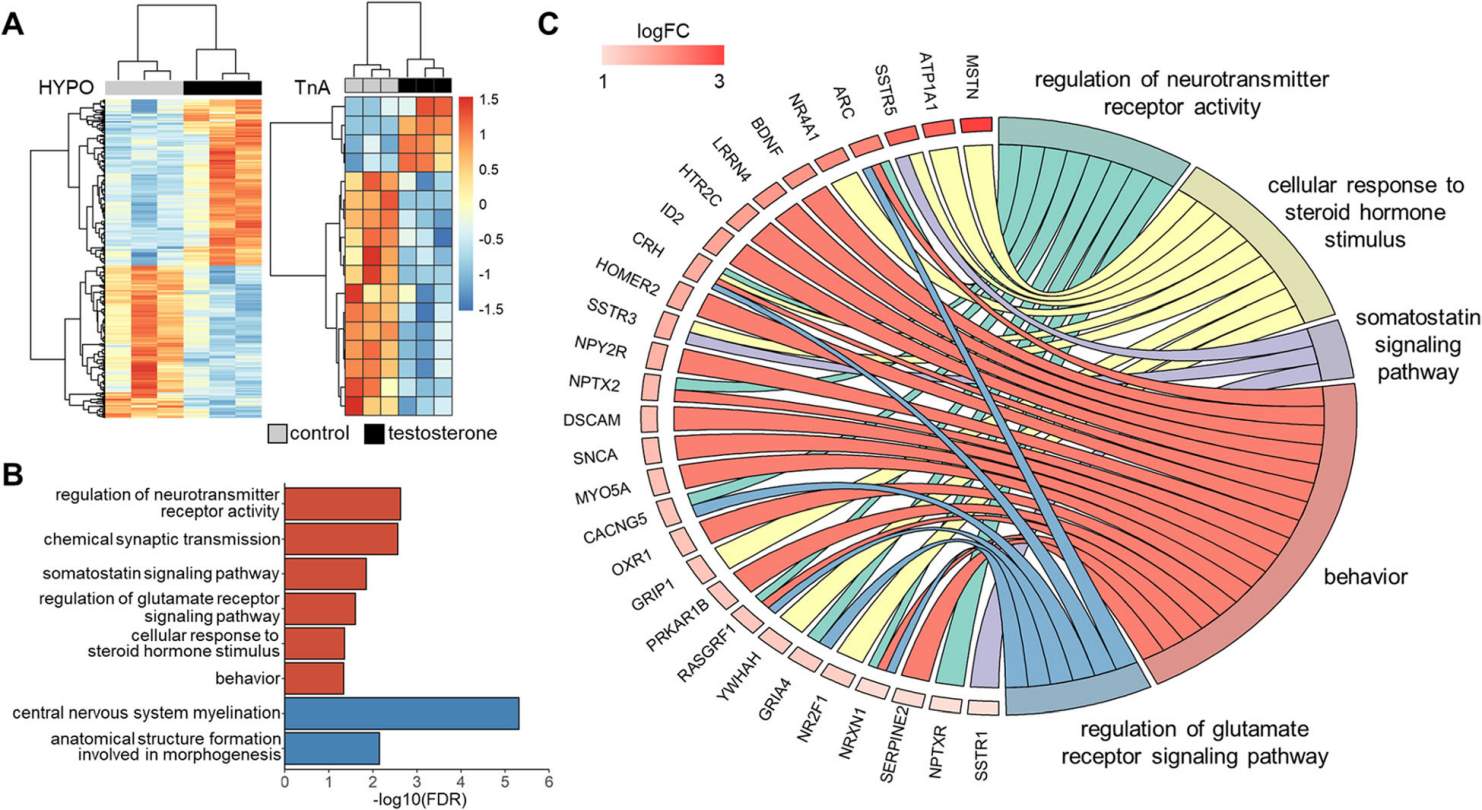
Differential expression and enrichment analyses. **a** Heatmaps depicting differentially expressed genes (DEGs) in the hypothalamus (HYPO) and nucleus taenia of the amygdala (TnA). Each column is an individual and each row is a gene. Color indicates normalized counts scaled across rows (red, higher expression; blue, lower expression). Clustered with a correlation distance measure. **b** Gene Ontology analysis showing the top 2 most significant biological processes among the up- (red) and down-regulated (blue) DEGs in the HYPO, along with aggression-related processes. **c** GOChord plot of DEGs in aggression-related processes. Genes are linked to assigned processes via colored ribbons and ordered according to log_2_ fold change (logFC), which is displayed next to genes. Descriptions of gene symbols can be found in Supplementary Table S1, Additional file 1

Our behavioral assays indicated that adult males from T-injected eggs were more aggressive than controls, and we next explored the genomic mechanisms potentially underlying this phenotypic divergence. RNA-seq was performed using the HYPO and TnA of males from T- and control-injected eggs (*n* = 3/treatment). We selected the males that were most responsive to the treatment (i.e., males from T-injected eggs that were unambiguously aggressive; Fig. 1) to minimize within-group variation that could have stemmed from variable sensitivities to the treatment. However, because treatment groups differed in their behaviors, we performed a permutational multivariate analysis of variance (PERMANOVA) which revealed significant differences in transcriptome-wide gene expression patterns in the HYPO due to prenatal treatment (*R*^2^ = 0.28, *F* = 1.71, *p* = 0.050), but not average aggression score (*R*^2^ = 0.24, *F* = 1.47, *p* = 0.167). This result suggests prenatal treatment is a better predictor of global gene expression patterns in the HYPO than individual-level differences in aggression. There were no significant differences due to either treatment (*R*^2^ = 0.15, *F* = 0.74, *p* = 0.649) or average aggression score (*R*^2^ = 0.25, *F* = 1.22, *p* = 0.303) in the TnA. Accordingly, the HYPO had the greatest number of differentially expressed genes (DEGs) between prenatal treatment groups. Males had 596 DEGs in the HYPO (*n* = 285 down-regulated genes, *n* = 311 up-regulated) and 17 in the TnA (*n* = 13 down-regulated, *n* = 4 up-regulated) (Fig. 2a). There were 4 DEGs shared between the brain regions, including down-regulation of a gene involved in tryptophan degradation (arylformidase; AFMID), up-regulation of a protein transport gene (golgin A2; GOLGA2), and differential expression of two additional uncharacterized genes. For the full list of DEGs see Supplementary Table S1, Additional file 1.

### Gene ontology enrichment analysis

We assessed whether any biological process Gene Ontology (GO) terms were over-represented in our list of DEGs. Up-regulated DEGs in the HYPO were most significantly enriched in regulation of neurotransmitter receptor activity (FDR = 0.002), while down-regulated DEGs were most significantly enriched in central nervous system myelination (FDR < 0.001) (Fig. 2b). DEGs enriched in terms that have been implicated in the regulation of aggression [14], including behavior, neurotransmitters (receptor activity and glutamate signaling), and hormones (steroids and somatostatin) are depicted in Fig. 2c. No enriched GO terms were found for DEGs in the TnA. For the full list of GO terms see Supplementary Table S2, Additional file 1.

### Protein-protein interaction network analysis

**Fig. 3:**
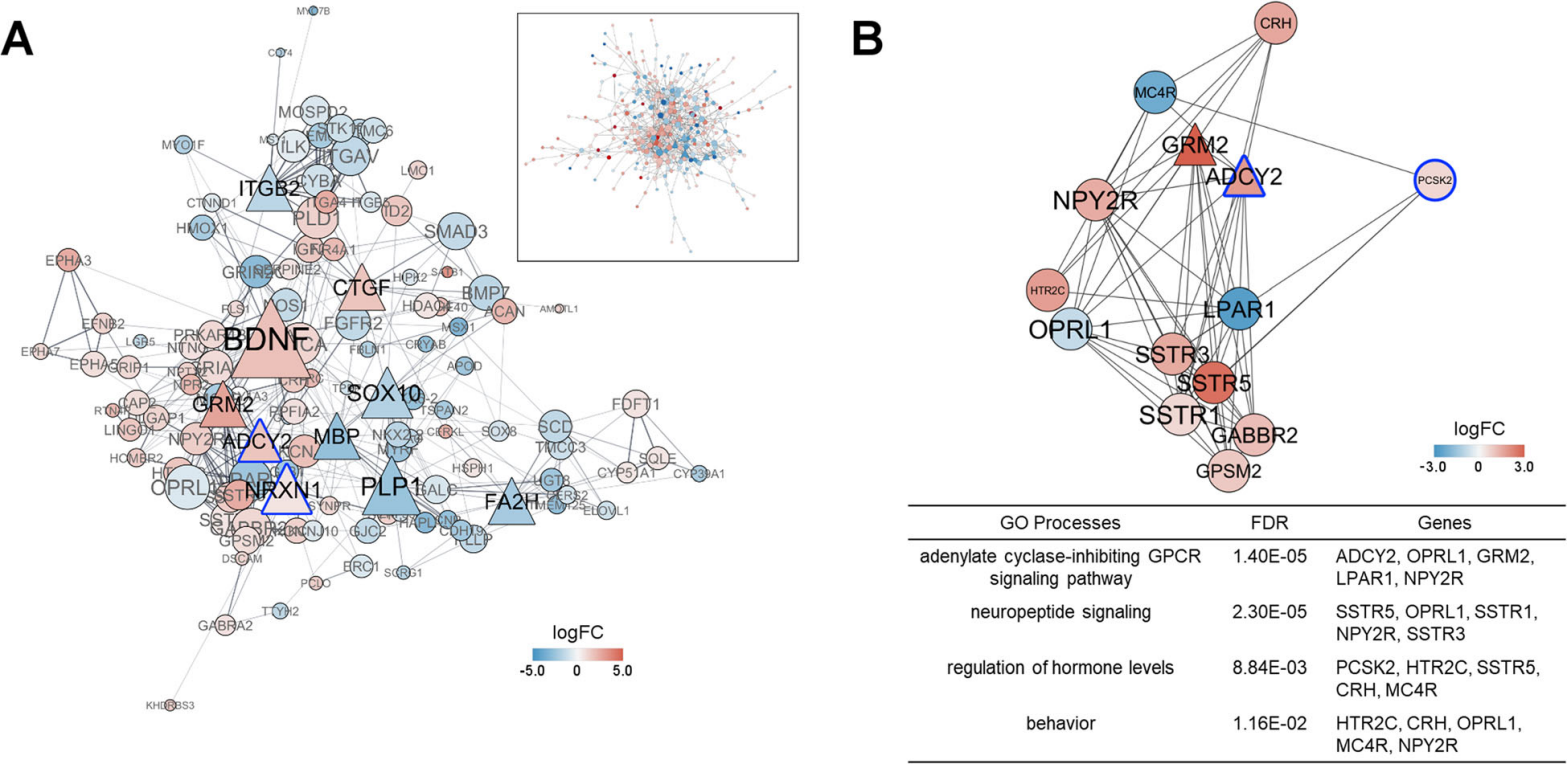
Protein-protein interaction (PPI) network analysis in the hypothalamus. **a** PPI network of differentially expressed genes; hub genes are shown in bold with their closest neighbors (inset depicts the full network). **b** PPI subnetwork and significant Gene Ontology (GO) processes. Node size represents number of degrees, triangular nodes indicate a potential hub gene, and color represents log_2_ fold change (logFC) (red, up-regulated; blue, down-regulated). Node border color, when present, indicates differential methylation (blue, lower methylation)

Based on the Search Tool for the Retrieval of Interacting Genes/Proteins (STRING) database, protein-protein interaction (PPI) networks were constructed using Cytoscape and the plug-ins cytoHubba and ClusterONE were used to identify important DEGs (i.e., potential hub genes) and subnetworks, respectively. For the DEGs in the HYPO, a network with 419 nodes and 787 edges was obtained (PPI enrichment: *p* < 0.001) and 10 potential hub genes were identified, including up-regulated DEGs in aggression-related GO terms, like brain-derived neurotrophic factor (BDNF, *n* = 31 degrees) and neurexin-1 (NRXN1, *n* = 19), as well as important signaling genes, like glutamate metabotropic receptor 2 (GRM2, *n* = 17) and adenylate cyclase 2 (ADCY2, *n* = 16) (Fig. 3a). Within the larger network, one subnetwork was identified containing 14 nodes (density = 0.66, *p* < 0.001; Fig. 3b). The GO processes enriched with the greatest number of DEGs in this subnetwork included G-protein-coupled receptor signaling (GPCR), neuropeptide signaling, regulation of hormone levels, and behavior (Supplementary Table S3, Additional file 1). A network with 13 nodes and 2 edges was obtained in the TnA, but it was not significant (PPI enrichment: *p* = 0.274).

### Identification of differentially methylated regions

**Fig. 4:**
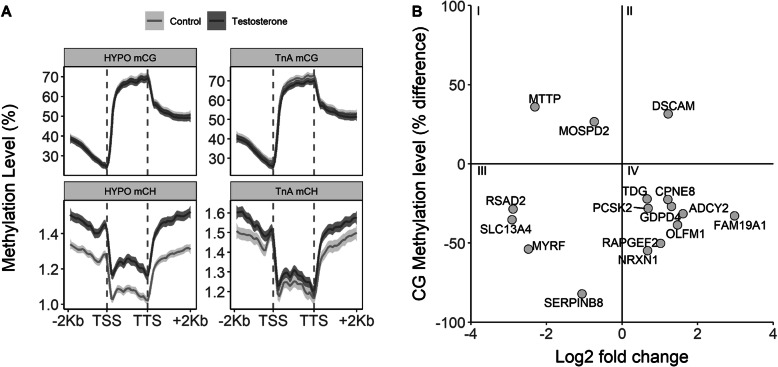
Differential methylation analyses. **a** Methylation patterns across genomic features in the hypothalamus (HYPO; left column) and nucleus taeniae of the amygdala (TnA; right column), including the 2 Kb region upstream of the transcription start site (TSS), gene body, and the 2 Kb region downstream of the transcription termination site (TTS). Shading indicates 95% CI. **b** Genes with differential expression and CG methylation in the HYPO. Genes in quadrants I and IV show an inverse pattern of expression and methylation. Descriptions of gene symbols can be found in Supplementary Table S7, Additional file 1

We performed MethylC-seq using the HYPO and TnA from the same males used for RNA-seq (*n* = 3/treatment). We first looked for large-scale differences in methylation levels between prenatal treatments. This was performed for both CpG (mCG) and non-CpG methylation (mCH, where H = A, C, or T), which is present in the neuronal genomes of birds [32]. Both mCG and mCH patterns were generally characterized by reduced methylation at the transcription start site (TSS), although mCH occurred at much lower levels (Fig. 4a). Levels of mCG and mCH did not significantly vary by prenatal treatment across genomic features in either tissue (gene bodies, *F*_1,4_ < 1.29, *p* > 0.320; 2 Kb upstream, *F*_1,4_ < 0.64, *p* > 0.468; 2 Kb downstream, *F*_1,4_ < 0.91, *p* > 0.394; Fig. 4a).

We next identified differentially methylated regions (DMRs) between prenatal treatments. Males had 1123 mCG DMRs in the HYPO, of which 468 (41.7%) were found within 2 Kb of a gene (*n* = 266 genes with hypomethylation, *n* = 125 genes with hypermethylation, and *n* = 11 genes with regions of both hypo- and hypermethylation), and 358 mCH DMRs, of which 138 (38.5%) were within 2 Kb of a gene (*n* = 13 genes with hypomethylation, *n* = 118 genes with hypermethylation). The TnA had 57 mCG DMRs, of which 27 (47.4%) were found within 2 Kb of a gene (*n* = 17 genes with hypomethylation, *n* = 8 genes with hypermethylation, *n* = 1 gene with regions of both hypo- and hypermethylation), and 15 mCH DMRs, of which 5 (33.3%) were found within 2 Kb of a gene (*n* = 2 genes with hypomethylation, *n* = 3 genes with hypermethylation). The majority of DMRs found within 2 Kb of a gene were located in the gene body, specifically introns, for both mCG and mCH (Supplementary Fig. S1, Additional file 2), similar to past methylation work in zebra finches [21]. For the full list of genes with DMRs see Supplementary Tables S4, S5, Additional file 1. A GO analysis of genes with mCG or mCH DMRs in each brain region indicated that genes with mCG DMRs in the HYPO were significantly enriched for processes like the regulation of molecular function (FDR = 0.01) and cell morphogenesis (FDR = 0.03; Supplementary Table S6, Additional file 1). No other enriched GO terms were found.

### Genes with differential methylation and expression

There were 16 DEGs in the HYPO that also had mCG DMRs (Fig. 4b; Supplementary Table S7, Additional file 1). No enriched GO terms were found among these genes; however, ADCY2 and prohormone convertase 2 (PCSK2) were identified as members of the PPI subnetwork (Fig. 3b) and DS cell adhesion molecule (DSCAM) and NRXN1 were highlighted in aggression-related GO terms (Fig. 2c). Eleven of the 16 genes showed inverse expression and methylation patterns (Fig. 4b). There were 4 DEGs in the male HYPO that also had mCH (Supplementary Table S7, Additional file 1). The TnA had no overlap between DEGs and DMRs.

## Discussion

Prenatal exposure to hormones like T can strongly influence developing offspring, even exerting long-term organizational effects. Accordingly, we show that adult males from T-injected eggs were more aggressive toward conspecific intruders than control males, similar to findings in other avian species [8]. Here, we examined a hypothesized mechanism by which prenatal T promotes this long-term behavioral change, including differential neural gene expression and methylation. We found hundreds of DEGs in the HYPO, but not the TnA, of males exposed to experimentally enhanced prenatal T. The TnA is involved in social arousal and sensory integration [29] and, as such, may play a larger role in more transient, socially activated genomic responses. Whereas the HYPO regulates upstream neuroendocrine processes associated with social behaviors [14, 30], perhaps making it better poised to generate lasting and far-reaching organizational effects. Many of the DEGs in the HYPO were associated with processes that have well-established links to the regulation of aggression [14]. Furthermore, we identified a highly connected PPI subnetwork in the HYPO that showed enrichment for signaling pathways and hormone regulation. Several DEGs in this subnetwork were also differentially methylated, suggesting prenatal T may generate epigenetic changes in key genes that are capable of influencing multiple aggression-related pathways.

DEGs in the HYPO were enriched for several aggression-related processes, including terms associated with behavior, steroids, glutamate, and somatostatin. One of the most frequently proposed mechanisms of action for the long-term behavioral effects of prenatal T are enhanced sex steroid production and/or sensitivity [12]. We found that plasma T levels did not differ between prenatal treatments, and while our power to detect statistical significance was low, this does agree with past work showing variable support for an effect of prenatal T on sex steroid production [10, 13]. Furthermore, we did not find that sex steroid receptors (e.g., androgen and estrogen receptors) were differentially expressed. However, the GO term cellular responses to steroid hormone stimulus was enriched with DEGs and this included nuclear transcription factors that interact with steroid receptors (e.g., NR2F1 and NR4A1), suggesting more subtle, nuanced changes may have occurred in the androgenic signaling system. Corticotropin releasing hormone (CRH) was also among the DEGs in this biological process, which could hint at differential regulation of corticosteroid secretion. Other up-regulated enriched processes included glutamate receptor and somatostatin signaling. Glutamate receptors excite neural circuits critical to aggressive behaviors and heightened expression may increase behavioral sensitivity, leading to more exaggerated responses [14]. Finally, we found that three somatostatin receptors (SSTR1, 3, 5) were up-regulated in males from T-injected eggs. This finding mirrors those in socially dominant fish in which somatostatin receptors are also expressed more highly [33–35], suggesting up-regulation of somatostatin signaling may be a conserved component of aggressive and socially dominant individuals. Collectively, these findings offer several candidate genes and pathways with well-established links to aggression that may be sensitive to prenatal T.

Epigenetic mechanisms are a promising candidate for explaining how long-term changes in gene expression are programmed. We found hundreds of gene-specific DMRs, both CpG and non-CpG, between treatment groups. A handful of genes with DMRs in the HYPO were also differentially expressed, suggestive of a regulatory relationship, although more extensive testing would be needed to validate this. Several of these genes were identified as being in a highly connected PPI subnetwork, including ADCY2 and PCSK2 (both up-regulated with decreased methylation in males from T-injected eggs). ADCY2 is a key enzyme in cAMP signaling that exerts a strong effect on gene transcription patterns [36]. PCSK2 is involved in processing numerous prohormones, including proopiomelanocortin, prosomatostatin, and proglucagon [37]. Our PPI analysis indicated that these genes may interact with somatostatin, corticotropin, glutamate, and melanocortin receptors involved in signaling and hormone regulation (Fig. 3b). Thus, prenatal T may cause altered DNA methylation of a few highly connected genes that have the potential to initiate a cascade of effects in numerous behavioral pathways. It is also possible that non-DEGs with DMRs are primed for future differential transcriptional responses (e.g., [38]). Genes with mCG in the HYPO were enriched for processes that could broadly influence neural activity, like cell morphogenesis. The mechanisms linking prenatal T and gene-specific methylation are currently unknown, but neurotransmitters and steroid receptors have shown the potential to direct methylation patterns via non-coding RNAs and DNA methyltransferase enzyme activity [39–41], altogether making this an exciting avenue for future research.

While both treatment groups were presented with the same social stimulus, T-treated males behaved more aggressively than controls and the subset we sampled for molecular analyses only included T males that showed consistently high aggression, making it possible that some of the genomic patterns we found are a result of acting aggressively. However, our data indicate we captured stable phenotypic differences resulting from the treatment rather than socially induced patterns. Transcriptome-wide patterns of gene expression in the HYPO were better explained by prenatal treatment than individual aggression scores. We also found expression profiles indicative of dominant behavioral phenotypes [33–35] rather than enrichment of the more labile neurogenomic processes associated with aggressive actions (e.g., energy metabolism [42]). Furthermore, evidence thus far indicates that socially induced changes in DNA methylation occur on longer time scales (hours compared to minutes) [43], suggesting the DMRs we identified are likely due to the prenatal treatment. Nevertheless, we are limited in our ability to form conclusions about within-group variation. While over two-thirds of males from T-treated eggs were more aggressive than the average control male, there were a handful that showed lower levels of aggression (Fig. 1), which highlights the need for future work to explore individual variation in sensitivity to prenatal hormones. Additionally, by having only sampled adults, we are also unable to separate whether the effects we observed were a direct effect of T on genes during embryonic development or if these changes in adulthood were due to more indirect effects (e.g., a consequence of altered juvenile development or changes in a subset of genes that then elicit downstream effects). Regardless, these data are an important first step that highlight molecular processes potentially affected by the cumulative phenotypic effects of prenatal T.

## Conclusions

We exposed male zebra finches to elevated prenatal T or a control and present data comparing adult aggressive behaviors and underlying neural gene expression and methylation patterns in two socially relevant brain regions. We found that adults from T-injected eggs showed increased aggressive behaviors along with enrichment of several aggression-related processes involving steroid, somatostatin, and glutamate signaling. Furthermore, there were two DEGs that were also differentially methylated, ADCY2 and PCSK2, that were identified as being highly connected genes within a subnetwork that showed enrichment for signaling pathways and hormone regulation. Thus, prenatal T may cause lasting changes in the methylation and expression of a few highly connected genes that have the potential to impact the expression of numerous aggression-related pathways. Collectively, these results highlight neurogenomic mechanisms that may play an important role in mediating the effects of prenatal T on long-term phenotypic outcomes.

## Methods

### Animal subject details

Male and female zebra finches from our breeding colony at the University of Georgia were randomly assigned in breeding pairs. Pairs were individually housed in standard cages (43 × 43 × 38 cm) with a light dark cycle of 14:10 h. They received two perches, a nest-box, and burlap ribbon as nesting material. They were provided with a mixed seed diet, water, and cuttlebone ad libitum. Food and water were checked and refreshed daily. Offspring were reared in the parental cage until their sexually dimorphic adult plumage was visible (~ 50 days post-hatch), at which point they were placed in same-sex flocks in standard cages. This study was approved by the University of Georgia’s Institutional Animal Care and Use Committee (AUP #A2014-03-014-Y2-A0).

### Prenatal hormone manipulation

We injected eggs from 20 breeding pairs with T (500 pg T in 5 μl peanut oil; Sigma Aldrich, cat. #T1500) or the control vehicle (5 μl peanut oil) on the morning eggs were laid (*n* = 109 eggs). This dose corresponds with the range of yolk androgens a female naturally allocates [44] and has been used to elicit phenotypic responses in zebra finch offspring in past work [45, 46]. The eggs were injected following the protocol in Winter et al. [31]. Briefly, eggs were held vertically with the rounded end pointing down and illuminated from beneath to visualize the yolk. Eggs were cleaned with 70% ethanol and injections were administered ~ 2 mm down from the pointed end of the egg at a 45^o^ angle using a sterile 10 μl Hamilton syringe. The hole was sealed with Loctite Ultra Gel Super Glue® and allowed to remain vertical for 10 min before being returned to the nest. We randomized the treatment assignment to the first egg in a clutch with subsequent eggs receiving alternating treatments. We performed a binomial generalized linear mixed model (GLMM) with hatching success as a binary response and evaluated whether treatment influenced hatchability, while controlling for natal nest as a random effect. We also included laying order as a variable as this can affect egg hormone levels [47]. We additionally recorded body mass (± 0.01 g) on the morning offspring hatched and used a LMM to test whether treatment or laying order affected this early condition metric, controlling for natal nest as a random effect. All mixed models were performed in R (version 3.5.2) with the lme4 package (version 1.1–25) [48].

### Aggression assay

We assayed aggression in 24 males (8 control and 16 T) once they reached sexual maturity, which occurs ~ 60 days post-hatch [49] (mean age = 138 days ±14 SE; mean mass = 15.02 g ± 0.35 SE). Aggression scores were assigned in individual conspecific intrusion trials. Subjects were isolated in a cage for 2 days to establish residency, after which a novel same-sex individual of similar mass (± 1.0 g) was placed in the subject’s cage for 15 min between 0700 and 1200 h. Observations to score aggression were carried out blindly with respect to treatment. The aggression score is the number of aggressive actions performed by the subject toward the intruder during the 15 min period. Aggressive actions included bill fencing (jab with bill), displacement (driving intruder off a perch), and chasing (following displaced bird) [50]. There were no instances in which the intruder aggressively attacked the resident. We performed two trials for each individual on separate days (~ 48 days apart ±5 SE) with novel intruders to determine repeatability of aggressive behaviors (proportion of variance accounted for by individual differences). We calculated repeatability using a poisson model for count data [51], while controlling for variation introduced by the treatment with the package rptR [52] in R (version 3.5.2). The model was run for 1000 bootstrap repeats. We used a LMM with natal nest ID as the random effect to determine if average aggression score was affected by treatment or laying order.

### Sample collections and dissections

At the completion of the behavioral assays, we collected three males from the T and control treatment groups to obtain trunk blood and brain tissue. While most males from T-treated eggs (68.8%) had an aggression score higher than the average control male, T males did show greater inter-individual variation in their behavioral responses (Fig. 1), which could have stemmed from variable sensitivities to the treatment. Therefore, as a first step toward understanding how prenatal T generates aggressive phenotypes, we minimized this within-group variability and selected males that were most sensitive to the treatment (i.e., males from T-injected eggs that had consistently high aggression scores), while also ensuring only unrelated males were selected to eliminate genetic bias (Fig. 1; Supplementary Fig. S2, Additional file 2). Selecting unambiguously aggressive males is not dissimilar to other studies that have used individuals with behavioral extremes to explore the molecular mechanisms underlying behavioral plasticity (e.g., [34, 53, 54]).

Following euthanasia by rapid decapitation, trunk blood was collected and kept on ice until whole blood could be centrifuged to collect plasma which was stored at -20 °C for hormone analysis. Immediately after trunk blood was collected, brains were rapidly removed, frozen in liquid nitrogen, and stored at -80 °C for later dissection. Euthanasia was performed without the use of anesthesia because decapitation is a very quick method of euthanasia for small animals [55] and anesthetic agents can alter brain activity [56]. To isolate tissues of interest in the brain, we followed the sample preparation guideline in the Songbird Neurogenomics (SoNG) Initiative [57]. Briefly, optimal cutting temperature compound was used to embed brains, 50 μm coronal sections were cut with a cryostat (Leica CM 3050S), and a sterile, disposable 1 mm biopsy punch (Integra, York, PA, USA) was used to make vertical punches to obtain the HYPO and TnA according to the songbird brain atlas [58] and ZEBrA database (Zebra Finch Expression Brain Atlas; http://www.zebrafinchatlas.org) (Supplementary Fig. S3, Additional file 2). The HYPO was identified using the third ventricle, optic chiasm, and ventral supraoptic decussation as landmarks. TnA was identified by following the robust nucleus of the arcopallium in towards the medial surface of the telencephalic lobe adjacent to the cerebellum. Tissue punches were immediately placed on dry ice and stored at -80 °C until further processing.

### Plasma testosterone assay

Hormones were extracted from plasma obtained from trunk blood using an ether extraction. T levels were determined using a radioimmunoassay following procedures described in Wingfield and Farner [59]. Average recovery was 92.9% (± 0.02 SE) and the intra-assay coefficient of variation was 3.2%. A t-test was used to determine if plasma T levels were affected by prenatal treatment. No random effect was included because no individuals were related.

### DNA/RNA extraction and sequencing

Tissues were homogenized with a Mini-BeadBeater-16 (BioSpec Products) in genomic lysis buffer to extract total DNA (Quick-gDNA MicroPrep kit, Zymo Research) or in TRIzol/TRI Reagent to extract total RNA (Direct-zol RNA MicroPrep kit, Zymo Research) with a DNase treatment step to remove DNA. The quality of extracted DNA and RNA was checked on an Agilent Bioanalyzer 2100. We used 500 ng of total RNA to construct stranded mRNA-Seq libraries using a KAPA Stranded mRNA-Seq Kit (Kapa Biosystems). Library preparation for whole-genome bisulfite sequencing was performed using 100 ng of DNA according to the MethylC-seq protocol in Urich et al. [60]. Library concentrations were checked with a Qubit 2.0 Fluorometer (Life Technologies) and size/quality was checked with a Fragment Analyzer (Advanced Analytical Technologies). RNA libraries were pooled and sequenced on two lanes of an Illumina NextSeq SE75 High Output Flow Cell. DNA libraries were run on one lane of an Illumina NextSeq PE150 High Output Flow Cell. Following initial analyses of mRNA expression, in which we discovered that HYPO was the most sensitive to the treatment, we ran HYPO samples on an additional lane of the NextSeq PE150 to improve coverage for methylome analyses. Sequencing was performed at the University of Georgia Genomics Facility.

### RNA-seq mapping and differential gene expression

RNA-seq data were mapped using STAR version 2.7.3a [61] with a 2-pass mapping approach to the *T. guttata* genome [62] version 3.2.4, downloaded from Ensembl (http://www.ensembl.org) in March 2017. Samples were first mapped individually, and splice junctions were identified. Samples were then remapped with splice junctions provided as input. Approx. 32.9 million reads/sample (± 2.2 SE) were mapped (Supplementary Table S8, Additional file 1). Read counts for protein coding genes were quantified using STAR and normalized values were determined using the DESeq2 package [63] in R/Bioconductor (genes with less than 10 total reads across samples were filtered).

We conducted a PERMANOVA based on Bray-Curtis distances of normalized counts (1000 permutations) using the adonis2 function in the R package vegan [64] to determine whether prenatal treatment or average aggression score explained significant variation in transcriptome-wide gene expression patterns in each tissue. We then performed a negative binomial generalized linear model with a local dispersion fit to determine differential gene expression within each tissue using DESeq2. Treatment was the fixed effect for each gene and we calculated per-gene Wald test statistics to identify significant differences. *P*-values were corrected using Benjamini-Hochberg corrections and FDR ≤ 0.10 (the default in DESeq2) were considered differentially expressed. Furthermore, because we had 3 biological replicates per treatment, we applied a log2 fold change cutoff of 0.5 (absolute value) based on recommendations in Schurch et al. [65].

### Gene ontology enrichment analysis

We performed a GO enrichment analysis to identify the biological processes over-represented in the DEGs found in each tissue. Up- and down-regulated DEGs were separately subjected to an over-representation analysis using PANTHER [66] with a Fisher’s Exact test and a cut-off of FDR ≤ 0.05 for statistical significance. The lists of GO terms were further summarized with REVIGO [67], which clusters GO terms based on semantic similarity (similarity threshold = 0.7).

### Protein-protein interaction network construction and analysis

Interaction associations of the proteins encoded by the DEGs were extracted from the STRING online database (https://string-db.org/) and were used to generate PPI networks of medium confidence (interaction scores > 0.40) for each tissue [68]. The genes in the PPI network are represented as nodes and the interactions between nodes are edges. Nodes with a high number of edges (aka degrees) are defined as hub genes that likely have highly influential biological functions. The Cytoscape plug-in CytoHubba was used to identify hub genes through degree method (top 10 genes with ≥10 degrees). We then identified subnetworks within PPI networks using ClusterONE, a Cytoscape plugin that finds densely connected regions that can be considered protein complexes. We only included significant (*p* < 0.05) subnetworks with a minimum of 10 nodes. A GO enrichment analysis was performed on DEGs in subnetworks.

### MethylC-seq mapping and differential methylation

Read trimming, alignment, and methylation calling were performed using the methylpy pipeline [69]. We sequenced TnA methylomes to ~ 5-7x coverage, sufficient to detect large-scale differences, and HYPO samples to ~ 16-24x coverage for more detailed analyses (Supplementary Table S9, Additional file 1). Non-conversion rates (the percentage of unmethyled cytosines that fail to be converted by bisulfite treatment) were determined by mapping reads from spiked in unmethylated Lambda phage DNA. All samples had a non-conversion rate between 0.17–0.22% (Supplementary Table S9, Additional file 1), indicating a high conversion from bisulfite treatment.

Methylation levels were determined by “weighted methylation”, which is the number of methylated cytosines divided by the total number of cytosines for a given region, weighting the data by sequence coverage [70]. This was performed for both CpG and non-CpG methylation. We used a LMM with adult ID as the random effect to determine if mCG or mCH levels differed between treatments within genomic features. We categorized genomic features using Ensembl (version 3.2.4), including 2 Kb upstream of the TSS (i.e., potential promoter) [21], gene body (exons and introns), and 2 Kb downstream of the transcription termination site (TTS).

We next looked for DMRs within 2 Kb of a gene body using the methylpy DMRfind function, as these were the DMRs we could confidently associate with genes. We filtered DMRs of low confidence (< 3 cytosines) and low coverage (missing data in ≥1 sample). We used a conservative significance cut-off of FDR ≤ 0.01 with minimally 20% methylation difference between the treatment groups for mCG and 1.3% for mCH. These treatment difference cut-offs are 1 SD from the mean for overall mCG and mCH. DEGs containing DMRs were identified and a GO enrichment analysis was performed on these genes.

## Supplementary Information

**Additional file 1: Table S1.** Differentially expressed genes between testosterone and control treatment groups. **Table S2.** Results of a gene ontology biological process statistical enrichment test for differentially expressed genes. **Table S3.** Results of a gene ontology statistical enrichment test for differentially expressed genes in the male hypothalamus protein-protein interaction subnetwork. **Table S4.** Differentially methylated CG regions between testosterone and control treatment groups. **Table S5.** Differentially methylated CH regions between testosterone and control treatment groups. **Table S6.** Results of a gene ontology statistical enrichment test for genes with differentially methylated CG regions in the male hypothalamus. **Table S7.** Genes with both differential methylation and mRNA expression between control and testosterone treatments. **Table S8.** RNA-Seq mapping statistics. **Table S9.** MethylC-seq mapping statistics.

**Additional file 2: Fig. S1.** Distribution of differentially methylated regions by treatment across genomic features in the hypothalamus and nucleus taenia of males. **Fig. S2.** Aggression scores by behavioral trial for males from eggs injected with testosterone or the control. **Fig. S3.** Schematic drawings of coronal sections through the zebra finch brain.

## Data Availability

Raw sequence reads can be found in the GEO database (GEO accession number GSE100396). Gene identifiers listed in Supplementary Tables correspond to the Ensembl database (http://www.ensembl.org). The datasets used in the current study are available in the supplementary files or will be made available from the corresponding author on request.

## Abbreviations

HYPO: Hypothalamus
TnA: Ventromedial telencephalon
PERMANOVA: Permutational multivariate analysis of variance
DEGs: Differentially expressed genes
DMRs: Differentially methylated regions
GO: Gene ontology
PPI: Protein-protein interaction
mCG: CpG methylation
mCH: Non-CpG methylation
TSS: Transcription start site
TTS: Transcription termination site
T: Testosterone
ADCY2: Adenylate cyclase 2
PCSK2: Proprotein convertase 2
AFMID: Arylformidase
GOLGA2: Golgin A2
BDNF: Brain derived neurotrophic factor
NRXN1: Neurexin-1
GRM2: Glutamate metabotropic receptor 2
STRING: Search Tool for the Retrieval of Interacting Genes/Proteins
DSCAM: DS cell adhesion molecule
SSTR: Somatostatin receptor
GPCR: G-protein-coupled receptor signaling
CRH: Corticotropin releasing hormone
RA: Robust nucleus of the arcopallium
