# Supplementary material for: Prenatal testosterone triggers long-term behavioral changes in male zebra finches: unravelling the neurogenomic mechanisms
Source: BMC Genomics. 2021 Mar 6;22:158. doi: 10.1186/s12864-021-07466-9 (PMC7937265; doi:10.1186/s12864-021-07466-9)
Supplement: Supplementary file 2 — Additional file 2: Fig. S1. Distribution of differentially methylated regions by treatment across genomic features in the hypothalamus and nucleus taenia of males. Fig. S2. Aggression scores by behavioral trial for males from eggs injected with testosterone or the control. Fig. S3. Schematic drawings of coronal sections through the zebra finch brain. [file 12864_2021_7466_MOESM2_ESM.docx]

**Supplemental Information for:**

Prenatal testosterone triggers long-term behavioral changes in male zebra finches: unravelling the neurogenomic mechanisms

Alexandra B. Bentz^1-3*^, Chad E. Niederhuth^4^, Laura L. Carruth^5^, and Kristen J. Navara^3^

^1^ Department of Biology, Indiana University, Bloomington, IN, 47405, USA

^2^ Center for the Integrative Study of Animal Behavior, Indiana University, Bloomington, IN 47405, USA

^3^ Poultry Science Department, University of Georgia, Athens, GA, 30602, USA

^4^ Department of Plant Biology, Michigan State University, East Lansing, MI, 48823, USA

^5^ Neuroscience Institute, Georgia State University, Atlanta, GA, 30303, USA


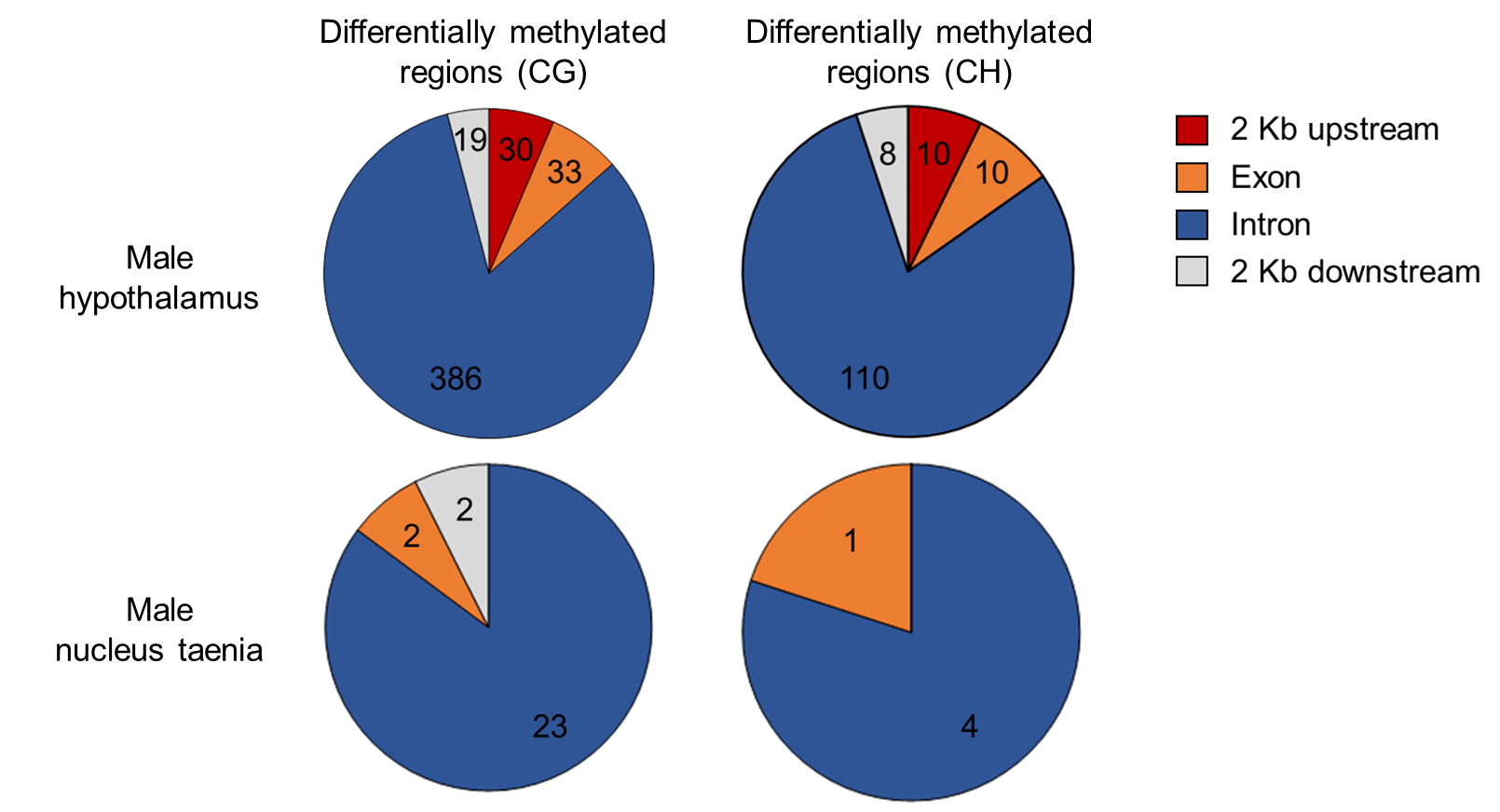


**Figure S1**. Distribution of CG (left column) and CH (right column) differentially methylated regions (DMRs) by treatment across genomic features in the hypothalamus and nucleus taenia of males. Color indicates genomic region and numbers represent how many DMRs were found.

**
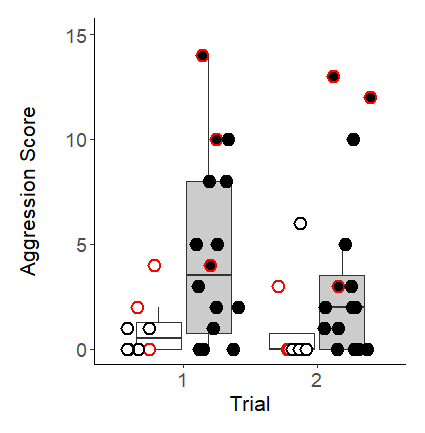
**

**Figure S2.** Aggression scores by behavioral trial for males from eggs injected with testosterone (T) (closed circles) or the control (open circles). Boxplots depict the median count (horizontal line) bounded by the upper and lower quartile of aggression scores for each treatment (males from T- and control-treated eggs are represented by gray and white, respectively), and whiskers represent 1.5 inter-quartile ranges. A red border identifies the three individuals per treatment that were collected for gene expression and methylation analyses.


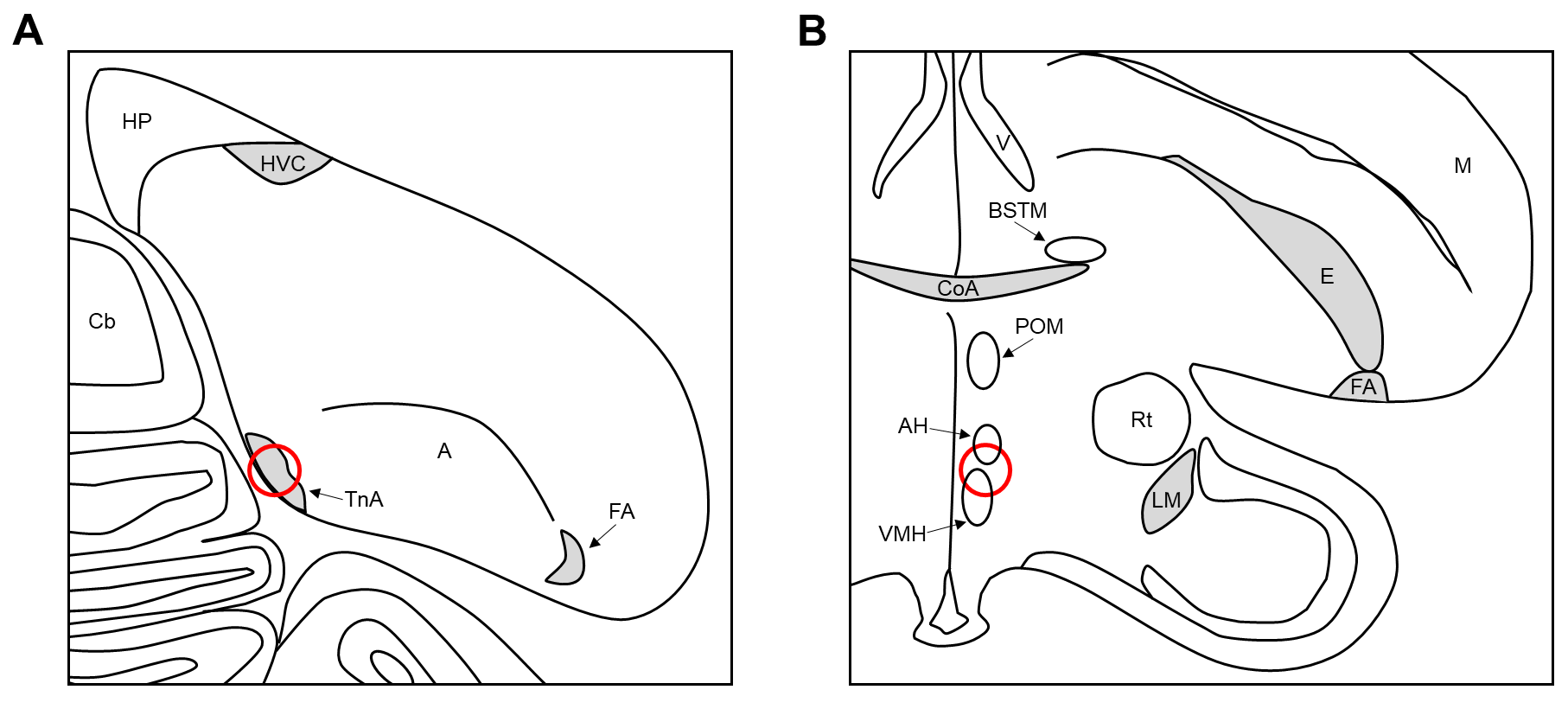


**Figure S3.** Schematic drawings of coronal sections through the zebra finch brain. Red circles indicate the region where tissues were sampled, including the (A) nucleus taenia of the amygdala (TnA) and (B) hypothalamus (consisting of anterior, AH, and ventromedial hypothalamus, VMH). Punches were made centrally in the TnA and ventrally in the hypothalamus to include AH and VMH and avoid the medial preoptic area (POM). Brain regions are drawn according to the songbird brain atlas [58] and ZEBrA database (Zebra Finch Expression Brain Atlas; http://www.zebrafinchatlas.org). Abbreviations: A, arcopallium; BSTM, medial part of the bed nucleus of the stria terminalis; Cb, cerebellum; CoA, anterior commissure; E, entopallium; FA, tractus fronto-arcopallialis; HP, hippocampus; HVC, acronym is name; LM, nucleus lentiformis mesencephali; M, mesopallium; Rt, nucleus rotundus; V, ventricle.
